# Deep fake detection using cascaded deep sparse auto-encoder for effective feature selection

**DOI:** 10.7717/peerj-cs.1040

**Published:** 2022-07-13

**Authors:** Saravana Balaji Balasubramanian, Jagadeesh Kannan R, Prabu P, Venkatachalam K, Pavel Trojovský

**Affiliations:** 1 Department of Information Technology, Lebanese French University, Erbil, Iraq; 2 School of Computer Science and Engineering, VIT Chennai, Chennai, Tamilnadu, India; 3 Department of Computer Science, CHRIST (Deemed to be University), Bangalore, Karnataka, India; 4 Department of Applied Cybernetics, University of Hradec Králové, Hradec Kralove, Czech Republic; 5 Department of Mathematics, University of Hradec Králové, Hradec Kralove, Czech Republic

**Keywords:** Deep fake detection, Deep learning, Deep sparse Auto encoder, Temporal Convolutional neural network, DNN, Face2Face, FaceSwap, Faceforensics++

## Abstract

In the recent research era, artificial intelligence techniques have been used for computer vision, big data analysis, and detection systems. The development of these advanced technologies has also increased security and privacy issues. One kind of this issue is Deepfakes which is the combined word of deep learning and fake. DeepFake refers to the formation of a fake image or video using artificial intelligence approaches which are created for political abuse, fake data transfer, and pornography. This paper has developed a Deepfake detection method by examining the computer vision features of the digital content. The computer vision features based on the frame change are extracted using a proposed deep learning model called the Cascaded Deep Sparse Auto Encoder (CDSAE) trained by temporal CNN. The detection process is performed using a Deep Neural Network (DNN) to classify the deep fake image/video from the real image/video. The proposed model is implemented using Face2Face, FaceSwap, and DFDC datasets which have secured an improved detection rate when compared to the traditional deep fake detection approaches.

## Introduction

Deepfake is represented as a forged image using deep learning algorithms. Deepfake research seems to be very interesting and significant for this digital era. The Internet has emerged with numerous multimedia content. Some intruders forge images and videos on social media. It affects in terms of individual reputation, rumors, political opinions, etc. Recent social media and networks are looking for intelligent algorithms to detect faked images and videos. Various problems such as image smoothing, edge preservation, filtering median are used in the forgery of original images (Luo et al., 2019; Pan et al., 2019b; Pan et al., 2019c). Sparse Auto-Encoder (SAE) works are based on concepts of artificial neural networks. SAE is an unsupervised machine learning principle used in difficult dimensionality reduction problems. Technically, it is widely used in feature selection and feature extraction with the backpropagation concept. So far, multimedia data are processed using steganalysis (Li et al., 2019), stegnography (Wang et al., 2019), water marking (Weng et al., 2019), multimedia coding (Stamm & Liu, 2011), anti-forensics countering image (Lei et al., 2016). Deep learning models based on CNN on five convolutional layers (Pan et al., 2019a) achieved good performance. The deblocking concept (Chen et al., 2015) suppresses JPEG artifacts and the filtered layer first, then the CNN concept is performed. Deep CNN computation perform better in forensic-based applications (Shan et al., 2019; Zhan et al., 2017; Bayar & Stamm, 2018; Chen et al., 2018a; Chen et al., 2018b). The above works motivate us to use the deep neural network in the detection of fake content for improved accuracy.

With respect to the development of recognition systems and algorithms, manipulating visual content technologies began to develop to substitute images and videos as accurate. There are many problems here with the trained adversarial generative network, and such deepfake content is challenging to identify and spread over the social network. This will invade the public’s privacy, including politicians, athletes, artists, and businesspeople. These deepfakes can damage the celebrity’s reputation, leading to financial and property losses, their professional careers, and also causes dramas in their personal lives. The political and news deepfakes can threaten the entire state, which lowers the trust between the authorities and citizens. Concerning the detection of deepfakes, the researchers focused on providing various solutions and published various scientific articles to recognize forgeries using deep neural networks. However, improving detection accuracy and system integrity is an issue that motivates us to develop a deep learning-based model for deepfake detection.

Computer vision with deep learning concepts opens the door for research to detect Deepfake using sparse autoencoders  (Güera & Delp, 2018). Autoencoders work better to distinguish fake manipulated frames in images and videos in Deepfake detection concepts. Today, even non-technical attackers can easily perform fake images by swapping the contents of videos and images. Deepfake creation minimizes trust among the public in reading digital media data. No one can ensure that the image shared is real or fake. A traditional detection technique does not use deep learning for detection. Deep learning creates a new research platform for fake detection in various applications and fields. The major contribution of this work is as follows

1. The input image and video frame data are preprocessed with Autoencoder’s dimensionality reduction approach.
2. Extraction of computer vision-based image features using proposed cascaded deep SAE trained by a Temporal Convolutional Neural Network for higher accuracy.
3. Real and deepfake images are classified using the Deep Neural Network (DNN).
4. Achievement of a better detection rate and improvement of the accuracy of a detection system using deep learning models.
5. Deep neural network for classifying CDSAE-based extracted computer vision features with high accuracy and less time.

The remaining section is formalized with five chapters. ‘Related Work’ consists of a review of the literature on Deepfake detection techniques. ‘Proposed CDSAE Methodology’ explains the algorithms and usage patterns of the proposed system. ‘Results and Discussions’ evaluates the results and the outcome. ‘Conclusion’ concludes the research with future directions.

## Related Work

This section presents a review of the literature on deepfake detection technologies, datasets, and algorithms.

This section presents a literature survey on deepfake detection technologies, datasets, and algorithms.  Güera & Delp (2018) developed a face swap detection model that focused on frames and regions of inconsistency using Long-Short-Term Memory (LSTM) and convolution neural network (CNN). For implementation, they used multiple website video datasets. Nguyen, Yamagishi & Echizen (2019) developed a capsule network to detect various types of replay attacks using recorded videos and printed images using a deep convolutional neural network. Xuan et al. (2019) proposed a deep preprocessing-based generalization model using DCGAN, WCGAN, and PGGAN with the Celeb-HQ dataset. This model learns more intrinsic features to classify real and fake images. Sabir et al. (2019) developed a deepfake detection model that focuses on temporal discrepancies using the Recurrent Neural Network with CNN to implement the FaceForensics++ dataset. They detected deepfake, face2face, and face swap from the video streams. Compared to existing approaches, it achieved improved accuracy of 4.55%.

Jeon, Bang & Woo (2020) introduced an image-oriented self-attention approach called Fine Tune Transformer that uses an attention model and a downsampling layer. This module is added with the pre-trained model to search for feature space to detect fake images. Deepfake-based datasets based on FDFtNEt and GAN-based were used for the experiments with an image resolution of 64 × 64. The proposed FDFtNet secured an accuracy of 90.29 for the detection of fake images and outperformed other approaches.  Jeon et al. (2020) developed a Transferable GAN image detection called T-GD for the detection of GAN images. It consists of a teacher and student model that is used to improve detection performance. The source dataset is trained with the teacher model, and then it is used as the starting point of the target data. The student model is trained by injecting the noise into the source and destination datasets with weight variations. T-GD has performed well on the source dataset by overcoming the catastrophic detection of GAN images with a small amount of data.

Hsu, Zhuang & Lee (2020) proposed a fake image detection based on deep learning using contrast loss. Initially, GAN was employed to generate fake and real image pairs. Then, the reduced denseness is developed to permit pairwise information with two streamed networks as input. The network is trained with pairwise learning to distinguish real and fake images. Finally, the classification layer is concatenated with the featured network to detect the input as a fake or accurate image. Their experimental evaluation states that other existing approaches outperform the proposed model.  Gandhi & Jain (2020) developed an adversarial network for deepfake image detections. The adversarial perturbations are created using the fast gradient sign method and the Carlini–Wagner *L*2 norm attack. The detector secured 95 accuracy on unperturbed deepfake using Lipschitz regularization and a deep image prior. Lipschitz increases the robustness of input perturbations. DIP removes the apprehension using generative CNN in an unsupervised way. Regularization improves perturbed deepfake detection with an average accuracy of 10 and boosts the black-box case. In the perturbed deepfake dataset, DIP secured 95 accuracy in the original detector and retained 98 accuracy in the 100 image subsample.

Wu et al. (2020) proposed an SSTNet for the detection of fake faces using Spatial, Stegananalysis, and Temporal features. Deep Neural Network was used to detect the spatial features with the finding of visual tampering, such as unnatural color, texture, and shape. Steganalysis features are extracted using convolutional filters. Temporal features were extracted using a recurrent neural network to distinguish the difference between consecutive frames. The SSTNet is tested against the GAN-based Deepfake dataset and the experimental results on Face Forensics++ dataset proves that SSTNet is superior to other existing approaches. Liu, Qi & Torr (2020) conducted a study on real and fake faces with two observations such as the fake face texture is different from the real one and global texture statistics are robust in image editing and transferable to fake faces from GAN and datasets. To motivate the observations, GramNet was developed for robust fake image detection using global image texture data. GramNet is robust in image editing, including downsampling, blur, JPEG compression, and noise.

Khalid & Woo (2020) proposed a deepfake detection model to overcome the problem of data scarcity and considered this as one-class anomaly detection. They developed OC-FakeDect using one class Variational Auto Encoder (VAE) which is used to train the real face images and detect the fake face images including deepfakes. This approach secured promising results in the detection of deepfakes by obtaining 97.5 accuracy on Neural Textures data and Face Forensics++ dataset.  Tariq, Lee & Woo (2021) proposed a convolutional LSTM with a Residual Network called CLRNet to detect the temporal data in Deepfakes. The proposed solution secured a better generalization in detecting deepfakes. They used Deepfake in the wild dataset for evaluation that collects videos from the Internet and has 150000 video frames. The detection precision was 93.86, which is significantly better than the existing approaches with defined margin.

Fung et al. (2021) designed a novel deepfake detection approach through unsupervised contrastive learning. They generate two versions of images and feed them as input to two sequential networks such as the encoder and projection head. This unsupervised model is further enhanced by training unsupervised features and linear classification networks. The experimental results show that unsupervised learning enables the detection performance of existing approaches in a dataset’s inter and intra settings. The list of papers mentioned above presents various challenges that need to be overcome in future research. Some techniques have performed overestimation in the feature selection. It increases both the testing and training time of the algorithm. Error rate also increases at the end of the result. Deep learning algorithms have performed underestimation in the selection of features. The major challenge is the overfitting problem which is the feature selection process. The proposed research addresses all of the above difficulties and focuses mainly on reducing the overfitting problem.

Table 1 presents the Deepfake detection survey in detail.

**Table 1:** Presents survey in detail of Deepfake detection.

Article	Dataset	Detection method	Research focus	Algorithm used
Güera & Delp (2018)	Multiple website video	FaceSwap detection model	Frames and inconsistency region	LSTM, CNN
Xuan et al. (2019)	celebA-HQ	Generalization	Deep preprocessing	DCGAN, WCGAN, PG-GAN
Sabir et al. (2019)	FF++	DeepFake, face2face, faceswap	Temporal discrepancies	RNN with CNN
Nguyen, Yamagishi & Echizen (2019)	FF, deepFake online, REPLAY-ATTACK DB.	Capsule in forensic application	Replay attacks, computer generated pictures	VGG, capsules
Hsu, Zhuang & Lee (2020)	CelebA, GAN-deepfake	Deepfake images	Pairewise learning	CNN concatenated with CFFA
Jeon et al. (2020)	TPGAN,Style GAN	Deepfake images	Self training	Resnet, efficientNet
Jeon et al. (2020)	CelebA, PGGAN, FF, DF	Deepfake images	Fine tune transformer	ResNetr, Xception, Squeeze Net.
Wu et al. (2020)	FF++ dataset	Deepfake images	Steganalysis feature	LSTM,XceptionNet
Gandhi & Jain (2020)	CelebA, GAN-deepfake	Deepfake tool	Adversarial perturbations	ResNet, VGG
Liu, Qi & Torr (2020)	FFHQ, CelebAHQ	Deepfake images	Texture analysis	Resnet
Khalid & Woo (2020)	FF++	Deepfake images	Variational autoencoder	Fake detection model
Tariq, Lee & Woo (2021)	FF++, DFD	Videos deepfake	Spatial images and temporal images	Convolutional LSTM model
Fung et al. (2021)	FF++,UADFV, CelebA-DF	Deepfake detection	Unsupervised learning	Xception, SVM, Bayes classification.

## Proposed CDSAE methodology

**Figure 1:**
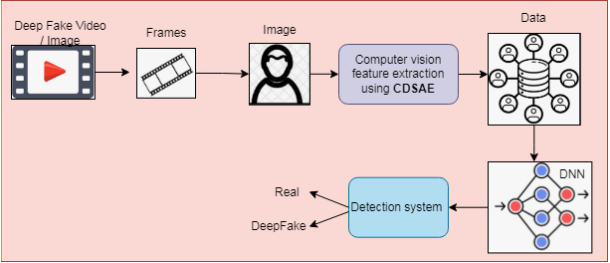
Proposed deepfake detection using computer vision features.

The detection process is implemented using a Deep Neural Network (DNN) with extracted features to classify regular and deepfake images or videos. The proposed deepfake detection system is shown in Fig. 1, which consists of two stages of processing, such as preprocessing and classification. In preprocessing phase, the input image or video is converted to frames for processing. From the frames, the facial part is detected using a Multi-task Cascaded Convolution Neural Network (Hinton & Salakhutdinov, 2006) and processed for the feature extraction and classification process. The computer vision features based on the frame change rate are extracted using the proposed deep learning feature selection method called the Cascaded Deep Sparse Auto Encoder (CDSAE), trained by temporal CNN (TCNN).

### Feature extraction using proposed cascaded deep sparse auto encoder trained by TCNN

**Figure 2:**
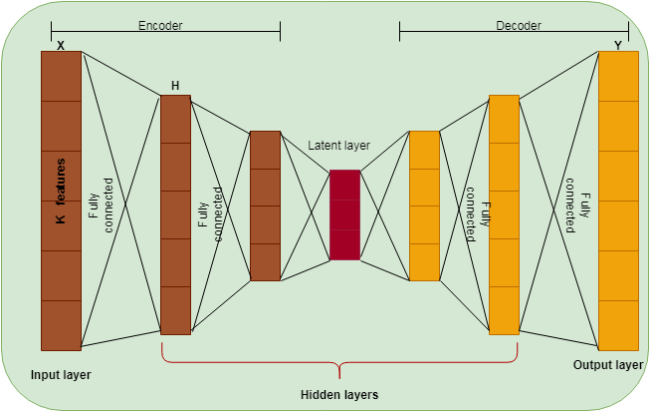
DSAE structure.

The normalized dataset is transformed into a reduced dimension using the Auto Encoder (AE) approach. This paper used traditional AE to reduce the dimensionality of the dataset and the version of the Cascaded Deep Sparse AE to select the computer vision features from the reduced dataset. AE is a deep learning-based neural network learned by an identity function. It is an unsupervised learning method widely used for dimensionality reduction (Hinton & Salakhutdinov, 2006). The feed-forward AE has been used, consisting of one layer with many nodes acting as a bottleneck. AE used backpropagation after the training process. In a bottleneck, the layer above is an encoder, and the layer below is called a decoder, as shown in Fig. 2. The input vector is transformed into a low-dimensional space in the encoder and reconstructed using the decoder. The hidden layer is denoted as *H*, *X* is the input, and *Y* is the reconstructed input. AE uses a linear activation function for a single hidden layer and a non-linear activation function for more than one hidden layer.

Deep AE provides better performance than traditional AE with an equal number of parameters (Lu et al., 2013; Maheswaran et al., 2015). The Deep AE is composed of many hidden layers, and the error is minimized using backpropagation compared to traditional AE which consists of one hidden layer with its input and output layer. Network performance is generalized by adding sparsity, which reduces network links. The sparse representation of classification has been studied by various researchers for face recognition (Gui et al., 2015; Maheswaran et al., 2017b), object categorization (Gui et al., 2014b), classification (Gui et al., 2014a) and regression oriented tasks (Bradski, 2000). Deep sparse AE is defined by two steps where the first step is to perform unsupervised layer wise greedy training as pre state. This step is used to extract the computer vision features that are more relevant with the help of unlabeled data in the form of encoder and decoder format. During this pre-training, the labeled data are not needed to reproduce the input to output. Let *X*_*p*_ be the input of the encoder which is encoded in the form of a function indicated in Eq. (1). The decoder decodes the encoded input as in Eq. (2) to reconstruct the real data input.

(1) \begin{eqnarray*}{E}_{p}=func({X}_{p}),\end{eqnarray*}

(2) \begin{eqnarray*}{D}_{p}=func({E}_{p}).\end{eqnarray*}

During pre-training, AE can reproduce the input to its respective output, which will lead to an overfitting issue. To handle overfitting, the sparsity term is added to the loss value function, which can generalize the training phase. The loss value function or the cost function is denoted in Eq. (3)

(3) \begin{eqnarray*}Loss=MSE+\alpha .{\tau }_{wt}+\beta \cdot ~{\tau }_{sty},\end{eqnarray*}

where *α* = coefficient of regression weight to prevent over-fitting,

*β* = parameter of sparsity regularization and penalty is set to the sparsity term

where *MSE* means square error between the decoded and real input defined as

(4) \begin{eqnarray*}MSE= \frac{1}{S} \sum _{i=1}^{I}\sum _{j=1}^{J}({{X}_{p}}_{ij}-{{D}_{p}}_{ij})^{2},\end{eqnarray*}

*H* is the number of hidden layers, *S* = number of input samples *τ*_*wt*_ is weight regularization with coefficient *α*, *τ*_*sty*_ is the sparsity regularization term with coefficient *β*:

(5) \begin{eqnarray*}{\tau }_{wt}= \frac{1}{2} \sum _{l=1}^{L}\sum _{i=1}^{I}\sum _{j=1}^{J}({W}_{ij}^{l})^{2},\end{eqnarray*}

(6) \begin{eqnarray*}{\tau }_{ty}=\sum _{k=1}^{{S}^{{^{\prime}}}}{PL}_{X} \left( \frac{\eta }{{\eta }_{k}} \right) =\sum _{k=1}^{{S}^{{^{\prime}}}} \left( \eta \cdot \log \nolimits \frac{\eta }{{\eta }_{k}} + \left( 1-\eta \right) \log \nolimits \frac{1-\eta }{1-{\eta }_{k}} \right) ,\end{eqnarray*}

where *I* is the number of instances, *J* is the number of variables, *W* are weights that control the weights of the network. *η*_*k*_ is an activation value of the neuron *k*, *η* is a desired activation value, *PL* sparsity proportion, *L* total number of layer and *s* total number of neurons.

**Figure 3:**
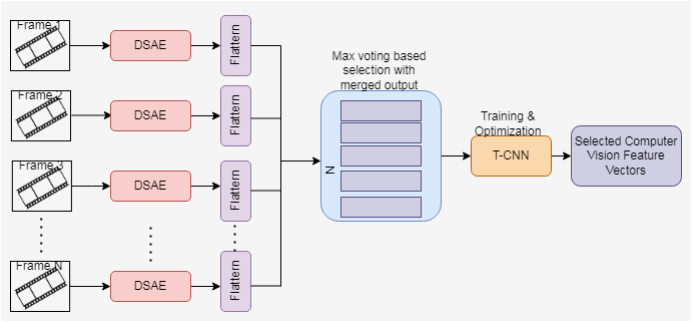
Proposed cascaded DSAE with TCNN-architecture.

For a selected neuron, if the desired and average activation function value is the same, then the sparsity regularization is zero. When the difference between *η* and *η*_*k*_ increases, then the sparsity regularization also increases. In DSAE, if the decoder is removed, then it becomes a deep network. The proposed computer vision-based feature extraction process using the Cascaded DSAE. The proposed architecture shown in Fig. 3 consists of the Cascaded DSAE each representing each frame. The output of multiple DSAEs is passed through the flatten layer, and the output is merged. Maximum voting is used to choose the best output from the merged results. The best features extracted through DSAE are trained by Temporal CNN to enhance the feature extraction process.

#### (a) CDSAE-T-CNN training and optimization

**Figure 4:**
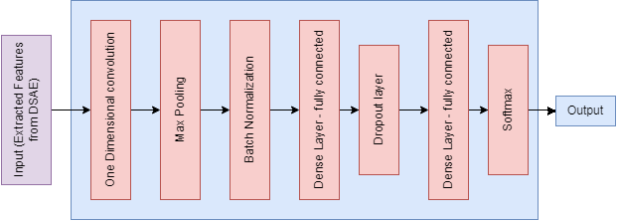
Temporal-CNN based optimization.

The proposed CDSAE is optimized and trained by T-CNN which consists of two one-dimensional convolution layers, two interconnected layers, and one softmax layer to perform the softmax function as shown in Fig. 4. For the overfitting issue, the maximum pooling, normalization, and dropout layers are used. The training and T-CNN based optimization is executed as follows;

1. One-dimensional convolution layer: it is used to comprise the feature input vectors and apply 64 various filters with a filter scale of 3.
2. Second-dimensional convolution layer: this works with 64 filters on four dimensions and learns the advanced functions until the pooling layer starts.
3. Maximum pooling: it chooses the filter that has secured the maximum value to avoid overfitting.
4. Batch Norm layer: it normalizes the data that are received from the previous layer.
5. Dense layer (fully connected): it has 140 intermediate nodes and 30% of dropout values.
6. Softmax layer: it generates two group; one for deepfake detection and the other for normal.

The fully connected layers at the beginning are responsible for estimating the output probability, as stated in Eq. (7) where *D* is the irregular event. This gate is changed to reflect the output as defined in Eq. (8)

(7) \begin{eqnarray*}P \left( {l}_{n}:{v}_{1:n} \right) =p({D}_{{l}_{n}}(H)),\end{eqnarray*}

(8) \begin{eqnarray*}{I}_{t}= \frac{1}{{e}_{t}} \sigma ({w}_{I}{X}_{t}+{V}_{i}{H}_{t-1}+{b}_{I}),\end{eqnarray*}

where *l*_*n*_ is the number of nodes in each layer, *e*_*t*_ is the type of event (in this study it is set to 2) and *σ* is the usual sigmoid activation function of a feature vector. The output of TCNN is generated as in Eq. (9)

(9) \begin{eqnarray*}{Y}_{t}=\sigma ({w}_{Y}{X}_{t}+{w}_{Y}{H}_{t-1}+{b}_{Y}),\end{eqnarray*}

where the parameters are defined as the previous sections *X*_*t*_ be the encoder input, *w* is weight, *H* is the number of hidden layers and *b* is the bias. Based on the number of layers, epochs, number of features, and nodes in each layer, the computational complexity of the proposed system is defined. Due to the implementation of the proposed deep learning model, the complexity of the proposed system is reduced. The extracted computer vision features are presented in Table 2.

**Table 2:** Extracted computer vision features using proposed CDSAE-TCNN.

No. of feature	Feature name	Description
1	MSE	Mean square error is the average variance between actual and estimated values.
2	PSNR	Peak signal to noise ratio is the ratio between maximum signal power and corrupted noise.
3	SSIM	Structural similarity index measure is the quality of cinematic and television pictures.
4	RGB	The percentage of image red, green and blue color value.
5	HSV	The percentage of image hue, saturation and value.
6	Histogram	Based on image brightness, it plots the no. of pixels in the image or frames.
7	Luminance	Total image brightness mean value.
8	Variance	Variance of image.
9	Edge-Density	Ration between edge pixel and total pixel of image.
10	DCT	Discrete Cosine transform: Image DCT bias value.

MSE is calculated using the intensity of two differences in the image pixels. PSNR aims to find the numerical difference rather than the visual variance of the human, since if MSE is zero, PSNR is also set as 0. SSIM has computed the temporal difference between luminance, contrast, and structure. R, G, B, and H, S, and v represent the color of the image. The distribution of the hue in the image is represented as a histogram. Average image brightness is represented as luminance. The brightness of the image variance is represented as a variance. Edge density is the ratio of the edge component of the pixel, and DCT is the sharpness of the image. Because the Deep Fake image synthesizes the target picture of each frame, it may lead to unnatural modifications in the computer vision features. When creating the deepfake, the target image should have limited resolution, and the size of the image has also changed. Therefore, inferior sharpness, blurring, and distortion occur in the deepfake, and the selection of these computer vision features should enhance the detection process.

### Detection using DNN

From the selected features, the variance is calculated based on the rate of change and is used for DNN learning. The dependent variable denotes the data as deepfake or real. The final output is computed as in Eq. (10)

(10) \begin{eqnarray*}{D}_{k}= \frac{1}{n} \sum _{i=1}^{n}({data}_{k,i}-\overline{dat{a}_{k}})^{2},\end{eqnarray*}

*D*_*k*_ is the value of the *k*th feature for the *i*th sample which is used for the training of DNN, *data*_*k*,*i*_ represents the *i*th data from prepossessing of the *k*th feature and $\overline{dat{a}_{k}}$ is the average of all data from feature extraction of the *k*th feature.

## Results and Discussions

The proposed CDSAE-TCNN and DNN for deep-fake detection is implemented using Open CV(Image Processing Library in Python) (Rossler et al., 2019) and for the DNN learning Keras module. DNN loss function is binary cross-entropy.

### Datasets used

The evaluation is performed using deepfake datasets such as Face2Face, FaceSwap developed by FaceForensics++ (Deepfake Detection Challenge, 2022) and DFDC(Deepfake Detection challenge) dataset from Kaggle (Zagoruyko & Komodakis, 2016). This dataset is comprised of 1000 videos and more. DFDC consists of more than 470GB of data. Additionally, the details of the datasets are presented in Table 3. The information in the dataset is related to genders, various races, and shooting circumstances. This proposed study has utilized 205 videos of Face2Face, 211 videos of FaceSwap, and 175 videos from DFDC datasets for experiment. Among the videos, there are 310 frames that are extracted from each video and the face of the frames that are extracted using MTCNN with the setting of 150 ×150 pixels. To extract the computer vision features from the frames, OpenCV has been used.

**Table 3:** Deepfake video image datasets used for proposed system.

Database	Total count of videos	Real video	No. of subjects	Fake video	Manipulation tool
FaceForensics++	3,000	1,000	–	5,000	FaceSwap Face2Face
DFDC	128,124	23,654	3,426	10,4500	DeepFake

### Hyper-parameter settings

The DNN hyperparameter and DNN have been fixed by choosing more than one optimizer and evaluating the proposed system. Table 4 shows the accuracy of various optimizers and the various numbers of hidden layers to fix the DNN hyperparameter. From the observation, the Adam optimizer has secured a lower loss and improved accuracy. Among the setting of hidden layers, five layers reach the improved accuracy. Therefore, the proposed model has been implemented with the setting of five hidden layers and Adam optimizer, which is used to handle the loss function.

### Evaluation

The proposed model is evaluated using evaluation metrics using the datasets and compared with ResNet (Sandler et al., 2018) and MobileNet (Schuldt, Laptev & Caputo, 2004; Maheswaran et al., 2021b) and SVM (Maheswaran et al., 2020b). Table 5 shows the comparative results on the accuracy of detection.

All methods perform well on Face2Face and FaceSwap datasets with a precision of 90% and more, except SVM. But for the DFDC dataset the performance methods have been reduced, and the minimum accuracy percentage has been obtained. Compared to the evaluation methods, the proposed feature selection approach has secured an improved accuracy of 98.7%, 98.5% and 97.63% for the Face2Face dataset, FaceSwap dataset, and DFDC datasets, respectively. The proposed model comparison based on content sharing performance is shown in Table 6. The comparison has revealed that the proposed system is superior in terms of structure, integrity, security, transparency, storage, and data sustainability.

**Table 4:** Proposed model performance to choose the hyperparameters.

Optimizer	No. of hidden layers	Loss	Accuracy
Stochastic Gradient Descent (SGD)	3	0.4561	73.2
	5	0.4281	76.9
	8	0.3971	79.8
AdaGrad	3	0.5612	71.8
	5	0.5109	78.4
	8	0.4713	81.2
Adam	3	0.1329	95.3
	5	0.0139	98.7
	8	0.1022	96.3

**Table 5:** Deepfake detection using the proposed model–performance comparison.

Methods	Datasets		
	Face2Face	FaceSwap	DFDC
Proposed CDSAE-DNN	98.7	98.5	97.63
ResNet	93.6	92.4	81.8
MobileNet	95.2	94.8	78.5
SVM	86.5	83.4	71.02

**Table 6:** Performance of proposed *vs* similar deepfake detection systems.

Methods	Decentralized structure	Data integrity	Security	Separated storage	Transparency	Data sustainability
Proposed CDSAE-DNN	✓	✓	✓	✓	✓	✓
ResNet	✓	✓	✓	×	✓	✓
MobileNet	✓	✓	✓	✓	✓	×
SVM	✓	×	✓	✓	✓	×

**Figure 5:**
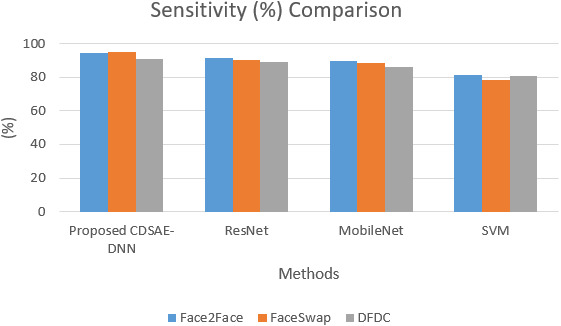
Sensitivity comparison of deepfake detection methods.

**Figure 6:**
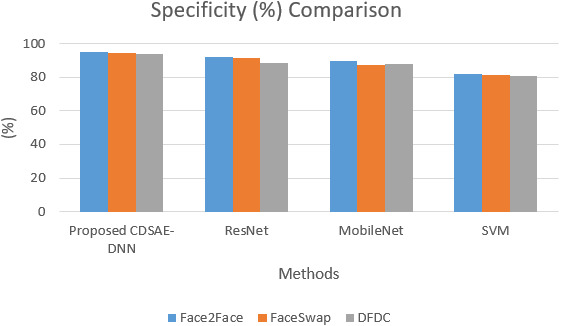
Specificity comparison of deepfake detection methods.

The sensitivity and specificity of the comparison for the proposed system is shown in Figs. 5 and 6. In addition to that, the graphical illustrations show that the improved percentage of sensitivity and specificity for all the datasets is obtained by the proposed model compared to the other similar deepfake detection systems. In addition to the proposed model, ResNet has performed well.

**Figure 7:**
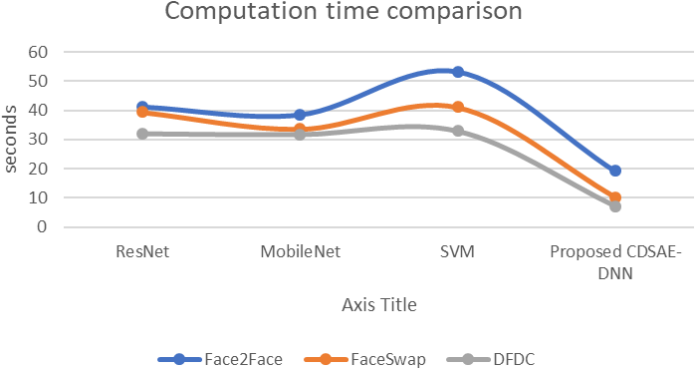
Computation time comparison.

**Figure 8:**
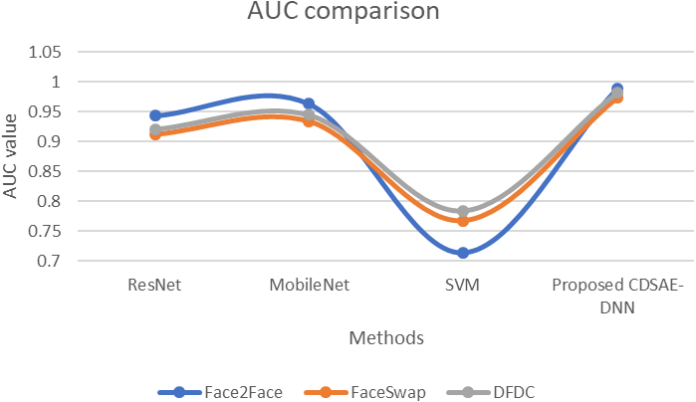
AUC comparison of proposed *vs* similar deepfake detection systems.

**Figure 9:**
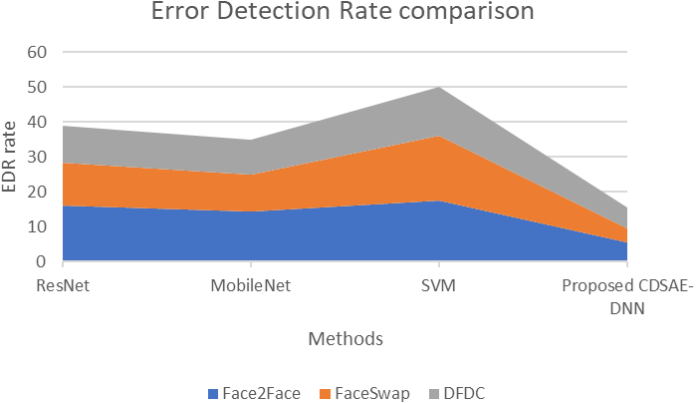
Error detection rate comparison of proposed *vs* similar deepfake detection systems.

The evaluation of the proposed system in terms of computation time, AUC, and error rate comparison are shown in Figs. 7, 8 and 9 using the deepfake datasets. From Fig. 7, for Face2Face dataset, the proposed model has utilized 19.2 s of time which is minimum than the other approaches such as ResNet, MobileNet and SVM which have secured 41.3 s, 38.6 s and 53.4 s respectively. For FaceSwap dataset, the proposed model has secured 10.2 s of time, which is minimal compared to other methods such as ResNet, MobileNet and SVM that have secured 39.4 s, 33.6 s, and 41.12 s sequentially. For the DFDC data set, the proposed model has secured 7.1 s, ResNet has secured 32.1 s, MobileNet has obtained 31.8 s and SVM has obtained 33.01 s. Compared to a similar deepfake detection system, the proposed model has obtained less computational time.

The evaluation of the proposed system in terms of AUC Fig. 8 using the deepfake datasets. For Face2Face dataset, the proposed model utilized 0.988 of time, which is improved than other approaches such as ResNet, MobileNet and SVM which secured 0.943, 0.963, and 0.714, respectively. For FaceSwap dataset, the proposed model secured 0.973 of time which is improved compared to the methods such as ResNet, MobileNet and SVM secured 0.912, 0.934 and 0.767 sequentially. For the DFDC dataset, the proposed model secured 0.982, ResNet secured 0.921, MobileNet obtained 0.945, and SVM obtained 0.784. Compared with similar deepfake detection systems, our proposed model obtained an improved AUC rate.

The proposed system evaluation in terms of the comparison of Error rate is shown in Fig. 9 using the deepfake datasets. From Fig. 9 the observations are as follows; for the Face2Face dataset, the proposed model has utilized 5.3 s of time, which is minimal compared to other approaches such as ResNet, MobileNet and SVM which have secured 15.9, 14.2 and 17.51 respectively. For FaceSwap dataset, the proposed model has secured 4.2 of time, which is minimum compared to the methods such as ResNet, MobileNet and SVM that have secured 12.3, 10.76 and 18.5 sequentially. For the DFDC dataset, the proposed model has secured 6.01, ResNet has secured 10.6, MobileNet has obtained 9.8 and SVM has obtained 14.02 respectively. Compared to similar deepfake detection systems, the proposed model has a lower error detection rate. Hence in the kind of evaluation, the proposed computer vision feature extraction based deepfake detection system has secured improved accuracy and efficiency on the detection of deepfake from normal video/image.

The fake detection that is performed by the proposed technique has a lower error rate compared to the existing techniques. Of the three datasets, the proposed CDSAE-DNN has a lower error in detecting fake faces in the input images.

## Conclusion

Deepfake detection in this research has used a new auto-encoder concept by cascading the data in a Deep sparse auto-encoder. Feature extraction performs better using the proposed deep learning model, which reduces over-fitness problems in deepfake detection. In addition, the concatenation of several classifiers improves detection performance. The outputs are selected multiple times to detect the best fake frame. It has achieved the best classification performance by multi-label classifiers performance. Input frames are studied deeply and superimposed single layer network operation is cascaded to find the best-faked frame.

In addition, the multilayer deep-sparse autoencoder reads multilayer inputs and performs the unsupervised function. Probability calculation helps to estimate the false image frame in the original setup. The experimental result has proved that the proposed algorithm computes 98 accuracy in less time. In the future, the concept of profound denoising can be used with DSAE to obtain better accuracy.

## Supplemental Information

10.7717/peerj-cs.1040/supp-1 Supplemental Information 1 Extracted computer vision features using proposed CDSAE-TCNN

10.7717/peerj-cs.1040/supp-2 Supplemental Information 2 Deep fake video image datasets used for proposed system

10.7717/peerj-cs.1040/supp-3 Supplemental Information 3 Proposed model performance to choose the hyperparameters

10.7717/peerj-cs.1040/supp-4 Supplemental Information 4 Deepfake detection using Proposed Model- Performance Comparison

10.7717/peerj-cs.1040/supp-5 Supplemental Information 5 Performance of proposed *vs* similar deep fake detection systems

10.7717/peerj-cs.1040/supp-6 Supplemental Information 6 Implementation

## Additional Information and Declarations

### Data Availability

The following information was supplied regarding data availability: The data is available at GitHub: https://github.com/NVlabs/ffhq-dataset.
