# Supplementary material for: Deep fake detection using cascaded deep sparse auto-encoder for effective feature selection
Source: PeerJ Comput Sci. 2022 Jul 13;8:e1040. doi: 10.7717/peerj-cs.1040 (PMC9299276; doi:10.7717/peerj-cs.1040)
Supplement: Supplemental Information 1 [file peerj-cs-08-1040-s001.docx]

Table 1: Extracted computer vision features using proposed CDSAE-TCNN

| Feature Number | Feature Name | Description |
| --- | --- | --- |
| 1 | MAP | Mean square error is the average variance between actual and estimated values |
| 2 | PSNR | Peak signal to noise ratio is the ratio between maximum signal power and corrupted noise |
| 3 | SIM | Structural similarity index measure is the quality of cinematic and television pictures |
| 4 | RGB | The percentage of image red, green and blue color value |
| 5 | HSV | The percentage of image hue, saturation, and value |
| 6 | Histogram | Based on image brightness, it plots the no. of pixels in the image or frames |
| 7 | Luminance | Total image brightness mean value |
| 8 | Variance | Variance of image |
| 9 | Edge-Density | The ratio between edge pixel and total pixel of the image |
| 10 | DCT | Discrete Cosine transform: Image DCT bias value |
