# Supplementary material for: Deep fake detection using cascaded deep sparse auto-encoder for effective feature selection
Source: PeerJ Comput Sci. 2022 Jul 13;8:e1040. doi: 10.7717/peerj-cs.1040 (PMC9299276; doi:10.7717/peerj-cs.1040)
Supplement: Supplemental Information 2 [file peerj-cs-08-1040-s002.docx]

Table 2: Deep fake video image datasets used for proposed system

| Database | Total count of videos | Real video | No. of Subjects | Fake video | Manipulation tool |
| --- | --- | --- | --- | --- | --- |
| FaceForensics++ | 3000 | 1000 | - | 5000 | FaceSwap  Face2Face |
| DFDC | 128124 | 23654 | 3426 | 104500 | DeepFake |
