# Supplementary material for: Deep fake detection using cascaded deep sparse auto-encoder for effective feature selection
Source: PeerJ Comput Sci. 2022 Jul 13;8:e1040. doi: 10.7717/peerj-cs.1040 (PMC9299276; doi:10.7717/peerj-cs.1040)
Supplement: Supplemental Information 3 [file peerj-cs-08-1040-s003.docx]

Table 3: Proposed model performance to choose the hyperparameters

| **Optimizer** | **No Hidden Layers** | **Loss** | **Accuracy** |
| --- | --- | --- | --- |
| Stochastic Gradient Descent (SGD) | 3 | 0.4561 | 73.2 |
|  | 5 | 0.4281 | 76.9 |
|  | 8 | 0.3971 | 79.8 |
| AdaGrad | 3 | 0.5612 | 71.8 |
|  | 5 | 0.5109 | 78.4 |
|  | 8 | 0.4713 | 81.2 |
| Adam | 3 | 0.1329 | 95.3 |
|  | **5** | **0.0139** | **98.7** |
|  | 8 | 0.1022 | 96.3 |
