# Supplementary material for: Deep fake detection using cascaded deep sparse auto-encoder for effective feature selection
Source: PeerJ Comput Sci. 2022 Jul 13;8:e1040. doi: 10.7717/peerj-cs.1040 (PMC9299276; doi:10.7717/peerj-cs.1040)
Supplement: Supplemental Information 4 [file peerj-cs-08-1040-s004.docx]

Table 4: Deepfake detection using Proposed Model- Performance Comparison

| **Methods** | **Datasets** | | |
| --- | --- | --- | --- |
|  | **Face2Face** | **FaceSwap** | **DFDC** |
| Proposed CDSAE-DNN | 98.7 | 98.5 | 97.63 |
| ResNet | 93.6 | 92.4 | 81.8 |
| MobileNet | 95.2 | 94.8 | 78.5 |
| SVM | 86.5 | 83.4 | 71.02 |
