# Supplementary material for: Deep fake detection using cascaded deep sparse auto-encoder for effective feature selection
Source: PeerJ Comput Sci. 2022 Jul 13;8:e1040. doi: 10.7717/peerj-cs.1040 (PMC9299276; doi:10.7717/peerj-cs.1040)
Supplement: Supplemental Information 5 [file peerj-cs-08-1040-s005.docx]

Table 5: Performance of proposed vs similar deep fake detection systems

| **Methods** | Decentralized Structure | Data Integrity | Security | Separated Storage | Transparency | Data Sustainability |
| --- | --- | --- | --- | --- | --- | --- |
| Proposed CDSAE-DNN | 🗸 | 🗸 | 🗸 | 🗸 | 🗸 | 🗸 |
| ResNet | 🗸 | 🗸 | 🗸 | $\times$ | 🗸 | 🗸 |
| MobileNet | 🗸 | 🗸 | 🗸 | 🗸 | 🗸 | $\times$ |
| SVM | 🗸 | $\times$ | 🗸 | 🗸 | 🗸 | $\times$ |
